# In Vitro Analysis of Human Immunodeficiency Virus Particle Dissociation: Gag Proteolytic Processing Influences Dissociation Kinetics

**DOI:** 10.1371/journal.pone.0099504

**Published:** 2014-06-10

**Authors:** Barbara Müller, Maria Anders, Jochen Reinstein

**Affiliations:** 1 Department of Infectious Diseases, Virology, University Hospital Heidelberg, Heidelberg, Germany; 2 Department of Biomolecular Mechanisms, Max Planck Institute for Medical Research, Heidelberg, Germany; Inserm, France

## Abstract

Human immunodeficiency virus particles undergo a step of proteolytic maturation, in which the main structural polyprotein Gag is cleaved into its mature subunits matrix (MA), capsid (CA), nucleocapsid (NC) and p6. Gag proteolytic processing is accompanied by a dramatic structural rearrangement within the virion, which is necessary for virus infectivity and has been proposed to proceed through a sequence of dissociation and reformation of the capsid lattice. Morphological maturation appears to be tightly regulated, with sequential cleavage events and two small spacer peptides within Gag playing important roles by regulating the disassembly of the immature capsid layer and formation of the mature capsid lattice. In order to measure the influence of individual Gag domains on lattice stability, we established Förster's resonance energy transfer (FRET) reporter virions and employed rapid kinetic FRET and light scatter measurements. This approach allowed us to measure dissociation properties of HIV-1 particles assembled in eukaryotic cells containing Gag proteins in different states of proteolytic processing. While the complex dissociation behavior of the particles prevented an assignment of kinetic rate constants to individual dissociation steps, our analyses revealed characteristic differences in the dissociation properties of the MA layer dependent on the presence of additional domains. The most striking effect observed here was a pronounced stabilization of the MA-CA layer mediated by the presence of the 14 amino acid long spacer peptide SP1 at the CA C-terminus, underlining the crucial role of this peptide for the resolution of the immature particle architecture.

## Introduction

Assembly and release of human immunodeficiency virus (HIV-1) progeny are orchestrated by the viral structural protein Gag [1], [2], which assembles at the plasma membrane of virus producing cells and recruits the viral RNA genome as well as other virion proteins and host cell factors to the viral budding site (reviewed in [2]). Gag represents the main constituent of viral particles, contributing ∼50% to total mass. HIV-1 Gag is initially synthesized as a polyprotein, consisting of the independently folded functional domains matrix (MA), capsid (CA), nucleocapsid (NC) and the C-terminal domain p6. In addition, two short spacer peptides, SP1 and SP2, separate the CA and NC domains, and the NC and p6 domains, respectively. A truncated sphere of ∼2500 Gag molecules arranged in a parallel fashion assembles at the cytoplasmic face of the plasma membrane to form the HIV-1 bud (reviewed in [1], [2]). Particles are released from the host cell by abscission of the surrounding lipid membrane mediated by the cellular endosomal sorting complex required for transport (ESCRT) machinery (reviewed in [2]). Concomitant with release, the Gag polyprotein is cleaved at five positions by the virus-encoded protease (PR), which separates the polyprotein into its functional domains and the spacer peptides. Proteolytic processing of Gag leads to a dramatic structural rearrangement of its subunits within the virion, with MA lining the lipid envelope of the mature particle and NC complexing and condensing the viral genome within a conical capsid assembled from CA subunits. This morphological maturation is a prerequisite for HIV-1 infectivity (reviewed in [2]) and presents HIV's solution to the ‘virus assembly-disassembly paradox’ [3]: a stable particulate structure assembled in the virus producing cell is subsequently converted into a metastable state, primed for disassembly in the next target cell. Accordingly, the immature HIV-1 Gag lattice was found to be stable upon detergent stripping of the surrounding lipid envelope, whereas mature particles readily dissociate under these conditions [4]–[6].

While the architectures of immature and mature virions are well characterized, the pathway of the morphological transition is currently not understood. In nascent buds and immature virions, Gag molecules are arranged into a hexameric lattice stabilized by lateral intermolecular interactions [7], [8]. Structural and mutational analyses located the most important intermolecular interactions within a region comprising the C-terminal domain of CA and the adjacent SP1 and demonstrated the crucial importance of this region for immature HIV-1 assembly [9], [10]. Hexamers of CA also form the basic building block of the mature HIV-1 capsid. However, the mature CA hexamer is structurally distinct from CA subunit hexamers within the immature Gag arrangement (reviewed in [1],[2],[11]) and surface residues predicted to be involved in immature and mature CA hexamers are largely non-overlapping [12]. With a center to center spacing of ∼8 nm, the immature lattice is also more tightly packed than the ∼10 nm mature lattice (reviewed in [1], [2], [11]) and only approximately half of all CA molecules contained in the particle are used for formation of the mature capsid [13]. These findings suggest that morphological maturation involves disassembly of the immature lattice followed by re-assembly of the mature capsid, rather than condensation of the semi-spherical immature CA layer into a conical core. Numerous studies indicate that this process is controlled through sequential processing of Gag mediated by different amino acid sequences at the PR cleavage sites. Apparently, the slow cleavage of SP1 and SP2 from their N-terminal partners CA and NC, respectively, plays an important regulatory role (reviewed in [1], [11]).

**Figure 1:**
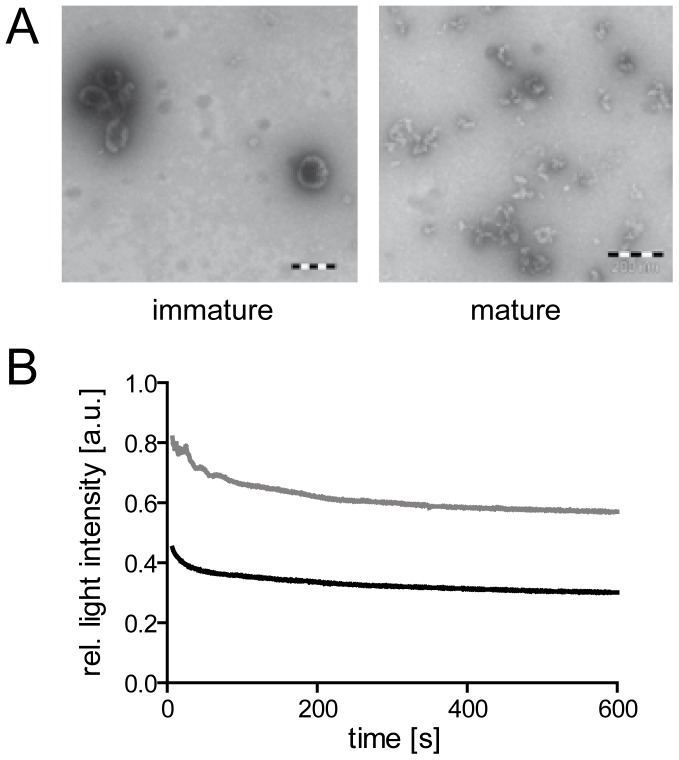
Effect of mild detergent treatment on virus particles. (**A**) Electron microscopy analysis of particle structures. Immature or mature particles, respectively, prepared as described in materials and methods were adhered to formvar coated electron microscopy grids and treated for 10 min with 0.05% TX-100 in PBS. Detergent solution was removed and protein structures were visualized by negative staining according to standard procedures. Scale bars: 200 nm. (**B**) Decay of light scattering signal upon detergent mediated particle dissociation at 25°C. Light scatter intensities of immature (gray line) and mature (black line) HIV-1 particle suspensions in PBS were determined using an SLM AB2 spectrofluorometer at a wavelength of 436 nm. At t = 0, TX-100 was added to a final concentration of 0.05%. Data shown are normalized to scatter intensities measured before detergent addition; raw data are presented as part of Figure S1.

Since controlled protein lattice dissociation appears to be a key feature of the HIV-1 maturation process, we wanted to measure dissociation rates of Gag assemblies at different stages of processing. Determination of association rates of purified recombinant HIV-1 CA *in vitro* by turbidity measurements [14] has proven valuable for analyses of inhibitory effects of CA subdomains, mutations in CA, inhibitory peptide derivatives or small molecules on the assembly process (e.g. [14]–[16]). A comparable system for measuring dissociation rates of Gag derived particles has not yet been established. We therefore decided to employ Förster's resonance energy transfer (FRET) reporter particles to analyze HIV-1 particle dissociation kinetics. Measurement of FRET [17], relying on the radiation-less transfer of energy between suitable fluorescence donor and acceptor pairs, is well established for analysis of intermolecular interactions *in vitro* or within cells in tissue culture. FRET occurs only between molecules in close spatial proximity and thus allows discrimination between protein complexes and monomeric subunits in solution. Various derivatives of HIV-1 Gag labeled with fluorescent proteins (FP) have been developed and characterized by us and others in earlier studies [18]–[21]. Gag derivatives labeled with suitable donor and acceptor FPs (*e.g*. enhanced cyan fluorescent protein, eCFP, and enhanced yellow fluorescent protein, eYFP) were previously used to analyze Gag oligomerization as well as the interaction of Gag with other proteins or with nucleic acids by FRET measurements in particle producing cells [22]–[25]. Here we have established a FRET based *in vitro* assay (Figure 1A) to monitor dissociation of mature, immature and partially processed HIV-1 virions released from particle producing cells with high time resolution and employed this system to compare dissociation kinetics of particles consisting of Gag proteins comprising different domains of the polyprotein precursor.

## Materials and Methods

### Plasmids and cell lines

Plasmids were based on the non-replication competent HIV provirus derivative pCHIV [26]. Sequences encoding for eCFP and eYFP, respectively, were inserted using a ClaI site introduced between the codons for amino acid 128 and 129 of HIV-1 MA, analogous to the previously described construct pCHIV^eGFP^ [26]. Mutations at individual PR recognition sites within Gag were introduced by transferring SphI/AgeI restriction fragments from pNLC4-3 based plasmids comprising point mutations at individual PR cleavage sites [27] into the context of pCHIV, pCHIV^eCFP^ and pCHIV^eYFP^, respectively. HEK 293T cells were grown in Dulbecco's modified Eagle's medium (GlutaMAX, Invitrogen) supplemented with penicillin (100 IU/ml), streptomycin (100 µg/ml) and 10% fetal calf serum (FCS) at 37°C, 5% CO_2_.

### Particle preparation

293T cells were transfected with the indicated plasmid derivatives using a standard polyethyleneimine transfection procedure. For the generation of single labeled particles (HIV^eCFP^ or HIV^eYFP^), equimolar mixtures of pCHIV and its labeled derivative (pCHIV^eCFP^ or pCHIV^eYFP^) were used. Dual-labeled FRET particles were generated by co-transfection of pCHIV, pCHIV^eCFP^ and pCHIV^eYFP^ or their derivatives carrying mutations at defined PR cleavage sites, respectively, at a molar ratio of 2∶1∶1. For production of immature particles, 2 µM lopinavir (LPV) was added to the tissue culture medium at the time of transfection. For specific inhibition of processing at the CA-SP1 cleavage site, 5 µM bevirimat (kindly provided by G. Kraus, Janssen Infectious Diseases-Diagnostics BVBA, Belgium) was added to the tissue culture medium at the time of transfection and replenished at 24 and 36 h post transfection (h.p.t.). At 44 h.p.t., tissue culture supernatant was harvested and cleared by passage through a 0.45 µm nitrocellulose filter. Particles were concentrated from the filtrate by ultracentrifugation through a 20% (w/w) sucrose cushion. Pelleted particles were resuspended in phosphate buffered saline (PBS) supplemented with 10 mM Hepes pH 7.5 and 10% FCS, distributed in aliquots, shock-frozen in liquid N_2_ and stored at −80°C.

### Quantitative Immunoblot analysis

Samples were separated by SDS-PAGE (17.5% acrylamide, acrylamide:bisacrylamide 200∶1) and transferred to a nitrocellulose membrane by semi-dry blotting. Membranes were blocked with 5% dried milk powder and incubated with polyclonal rabbit antisera raised against recombinant HIV-1 MA or CA, respectively. Bound antibodies were detected by quantitative immunoblot (LiCor Odyssey), using secondary antibodies, protocols and software provided by the instrument manufacturer. For quantitation of particle amounts, purified recombinant HIV-1 CA protein was analyzed in parallel.

### Fluorescence measurements

Recording of spectra and slow time scale kinetic measurements were performed using an SLM Aminco AB-2 spectrophotometer. Samples were diluted in PBS to a final volume of 100 µl and incubated at 25°C. Spectra were recorded using an excitation wavelength of 433 nm. For time resolved measurements, fluorescence emission at 528 nm was recorded with a time resolution of 1 s. Fluorescence of the sample was recorded over 1 min to obtain the value corresponding to 100% relative FRET intensity. Subsequently, TritonX-100 (TX-100; Merck) was added to the cuvette to a final concentration of 0.05% (v/v), samples were mixed by manual pipetting and measurement of fluorescence intensity was continued at ∼4 s after addition of detergent. Values were volume corrected and normalized to the initial fluorescence before detergent addition. Data were analyzed using GraphPad Prism.

### Protease treatment

Suspensions of HIV^eCFP/eYFP^ particles in PBS dissociated by addition of 0.05% TX-100 (v/v) were treated with recombinant HIV-1 PR under continuous spectroscopic observation. The reaction was carried out at neutral pH (7.2) in order to avoid quenching of the fluorophores, resulting in sub-optimal buffer conditions for HIV-1 PR (optimum for Gag cleavage *in vitro* pH∼6.0, [28]). This was compensated for by a high enzyme concentration. FRET reporter particles suspended in PBS were treated with 0.05% TX-100 and incubated for 500 s at 25°C to allow for particle dissociation. Purified recombinant HIV-1 PR (Ascoprot Biotech, Prague, Czech Republic; final concentration 18.5 nM) was added and incubation was continued. Samples were excited at a wavelength of 433 nm and emission at 528 nm was measured over time with a resolution of 1 s.

### Stopped flow measurements

Fast kinetics were recorded with a SF-61 DX2 Double Mixing Stopped-Flow apparatus (TgK Scientific Limited, Bradford-on-Avon; GB). All experiments were carried out in PBS at 25°C. TX-100 was added when indicated to a final concentration of 0.05% (v/v). In order to ensure that the elevated flow rates accompanying the automated mixing procedure did not lead to disruption of particles through shearing forces, experiments were carried out at lower pressure (2.5 bar). The resulting lower mixing velocities led to a slightly increased dead time of the instrument (∼4 ms, instead of 1.5 ms at 5 bar). For simultaneous measurement of eCFP/eYFP FRET signal and static light scattering samples were excited at a wavelength of 436 nm and slit width of 4 nm. FRET signal emission and static light scattering was monitored with photo multipliers and a long pass filter (515FG05, FRET) or band pass filter (440FS10, SLS), respectively. Kinetic traces were recorded on a logarithmic time scale over 100 s or 300 s, respectively. Five to six traces per sample were routinely averaged for each analysis and fitted using either the manufacturer's software package (Kinetic Studio Version 2.23) or GraphPad Prism software.

## Results and Discussion

In order to analyze HIV-1 particle dissociation properties, we first established the conditions for viral envelope disruption by mild detergent treatment. Earlier biochemical and electron microscopy analyses had shown that mature HIV-1 virions readily dissociate upon detergent mediated removal of the lipid envelope, while the Gag protein shell of immature particles is resistant to detergent addition [4]–[6]. Accordingly, qualitative assessment by negative stain electron microscopy confirmed that brief incubation of purified mature HIV-1 particles with 0.05% TritonX-100 (TX-100) resulted in disruption of detectable virus-like structures, while structures resembling intact Gag shells were still observed in the case of immature virions (Figure 1A). To obtain time resolved information, the effect of detergent on suspensions of immature and mature particles, respectively, was determined by static light scatter analyses. A suspension of HIV-1 particles in PBS yielded an approximately 10fold stronger light scatter signal, compared to a ‘mock particle’ sample prepared in parallel from cells transfected with empty vector (Figure S1). Disruption of the viral envelope by addition of detergent led to scatter signal decay within approximately 30 s. The amplitude of signal intensity loss showed a characteristic difference, however, consistently reaching values corresponding to ∼60% or 35% of the original value for immature and mature particles, respectively (Figure 1B, Figure S1 and data not shown). While these control measurements indicated the overall time frame of dissociation and revealed a qualitative difference between mature and immature particles, they did not provide kinetic information. The time traces recorded were complex, comprising at least one rapid phase that could not be captured with a manual mixing and measurement setup. Furthermore, virus samples derived from the tissue culture supernatant of particle producing cells may contain significant amounts of non-viral vesicles and particulate material [29], [30], confounding data interpretation. Therefore, a FRET-based setup was chosen to allow specific measurements of Gag-Gag interactions.

**Figure 2:**
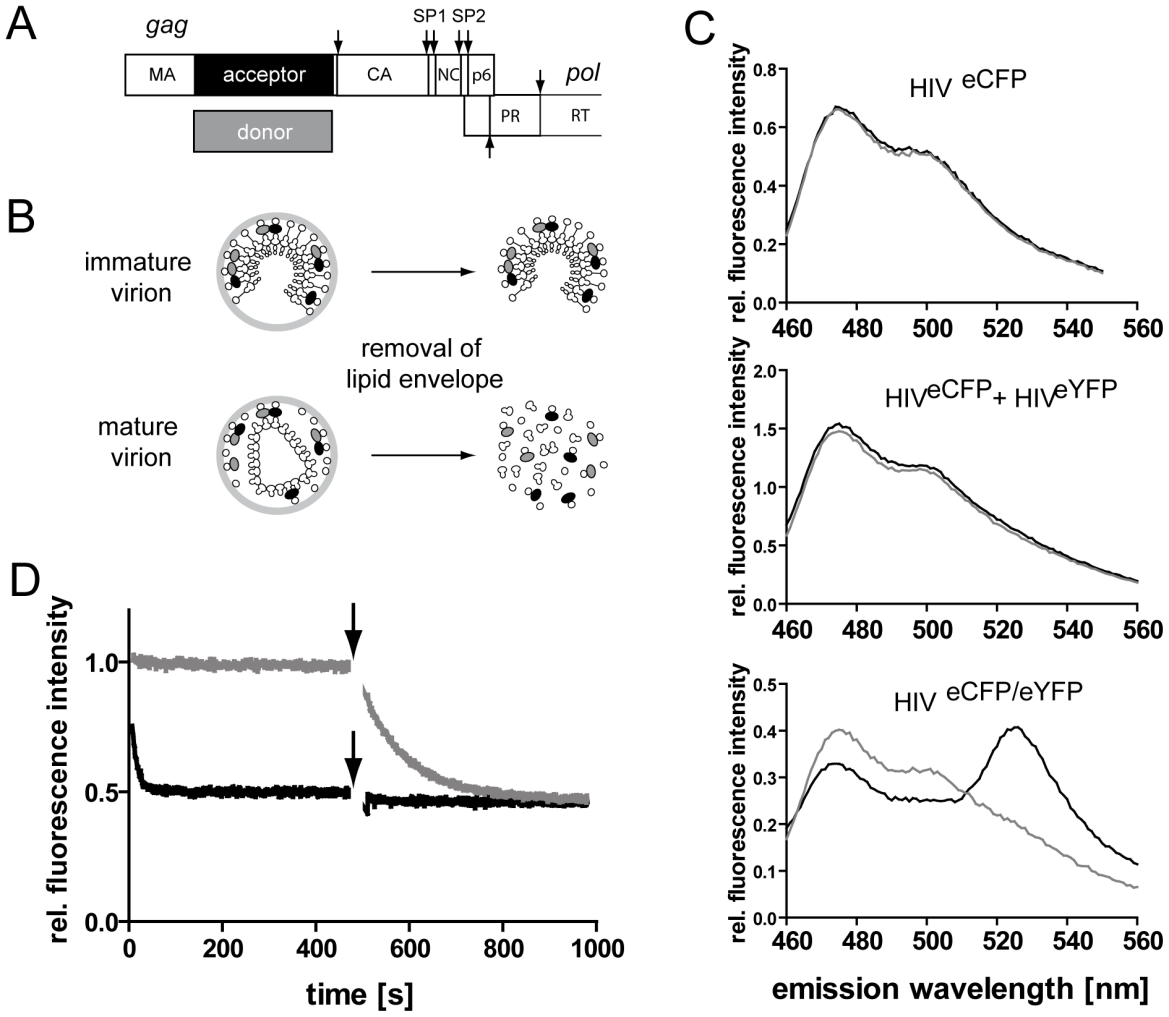
FRET based measurements of HIV particle dissociation. (**A**) Schematic representation of the HIV-1 Gag protein, showing the position of the insertion of the FRET donor or acceptor fluorophore. Arrows indicate cleavage sites for the viral protease. (**B**) HIV^eCFP/eYFP^ reporter particles carrying a mixture of untagged, donor- and acceptor-tagged Gag polyproteins. Detergent treatment of mature particles disrupts the lipid envelope of the virion, resulting in dissociation of the labeled MA layer and the viral core. (**C**) Emission spectra obtained for single labeled and dual labeled fluorescent HIV particles. Particles were purified from the tissue culture supernatant of 293T cells transfected with an equimolar mixture of pCHIV and pCHIV^eCFP^ (HIV^eCFP^), pCHIV and pCHIV^eYFP^ (HIV^eYFP^) or with pCHIV, pCHIV^eCFP^ and pCHIV^eYFP^ at a molar ratio 2∶1∶1, (HIV^eCFP/eYFP^), respectively. Purified particles were incubated in PBS at 25°C, excited at 433 nm and emission spectra were recorded (black lines).Subsequently, TX-100 was added to a final concentration of 0.05% to disrupt the viral envelope and emission spectra were again recorded after 15 min incubation (gray lines). (**D**) Time dependence of FRET intensity changes upon disruption of the lipid envelope. Mature (black line) or immature (gray line) HIV^eCFP/eYFP^ particles were incubated in PBS at 25°C and fluorescence intensity was recorded at 433 nm/528 nm. At t = 0 s, 0.05% TX-100 was added and incubation was continued. At t = 500 s (arrows), purified HIV-1 PR was added and incubation was continued. Intensity values shown are normalized to the intensity measured before detergent addition.

For the generation of HIV-1 FRET reporter particles we made use of a previously described virus derivative carrying a fluorescent protein (FP) as an additional domain between the MA and CA domains of Gag in the viral context (Figure 2A) [20]. Here, the FP moiety was inserted upstream of the PR recognition site separating MA and CA and remained fused to the MA protein after proteolytic maturation of the virion. Mixed particles carrying ∼50% labeled Gag molecules can be obtained by co-expression with wild-type virus; these particles display wild-type morphology and entry efficiency [20]. In order to obtain particles suitable for FRET analyses, the eGFP moiety was replaced by a fluorescence donor (eCFP) or fluorescence acceptor (eYFP), respectively. Thereby donor and acceptor molecules are positioned between the MA and CA domains of the Gag shell in immature particles, or at the C-terminus of the membrane associated MA protein in the mature virion, respectively (Figure 2 A, B). Constructs were generated in the context of the subviral plasmid pCHIV, which directs expression of all HIV-1_NL4-3_ proteins with the exception of Nef, but is not replication competent due to the lack of both viral long terminal repeat regions [26]. Particles containing a mixture of approximately 25% eCFP-tagged, 25% eYFP-tagged and 50% unlabeled Gag were prepared from particle producing 293T cells. Although this approach does not allow to precisely control the relative amount of fluorescence donor and acceptor molecules in each individual particle, fluorescence microscopy analyses confirmed that it leads to efficient formation of double-labeled virions (Figure S2). Particles were diluted in phosphate buffered saline (PBS) or in PBS containing 0.05% TX-100, respectively, and emission spectra were recorded (Figure 2C). Virions containing only unlabeled and eCFP-labeled MA (HIV^eCFP^) displayed an emission spectrum characteristic for eCFP upon excitation at 433 nm. Addition of separately prepared eYFP-labeled particles (HIV^eYFP^) to the measurement did not influence the spectrum recorded for HIV^eCFP^ and detergent-induced disruption of the viral envelope did not alter emission spectra derived from HIV^eYFP^ alone or from a mixture of separately prepared HIV^eCFP^ and HIV^eYFP^ particles, respectively. In contrast, FRET reporter particles carrying a mixture of unlabeled, eCFP-labeled and eYFP-labeled MA (HIV^eCFP/eYFP^) displayed a distinct emission maximum at 528 nm, indicative of FRET between eCFP and eYFP. In contrast, the emission spectrum of particles treated with 0.05% TX-100 closely resembled that of HIV^eCFP^ alone, indicating that detergent stripping of particles resulted in nearly complete dissociation of MA.eCFP/MA.eYFP complexes (Figure 2C).

The FRET reporter system was employed to investigate the time dependence of HIV particle dissociation *in vitro*. HIV^eCFP/eYFP^ particles were incubated at 25°C and fluorescence emission at 528 nm was recorded using an excitation wavelength of 433 nm with a time resolution of 1 s (Figure 2D, black line). Addition of detergent resulted in an exponential decay of the FRET specific signal over a period of 1–2 minutes. Observed kinetics were independent from the detergent concentration (Figure S3), indicating that TX-100 did not directly contribute to disruption of protein-protein interactions. In the case of immature HIV^eCFP/eYFP^ particles, detergent addition resulted in only a very minor decrease of the fluorescence signal (Figure 2D, gray line). To verify that the difference in FRET signal intensity change observed between mature and immature particles depended on Gag maturation status, purified recombinant HIV-1 PR was added to the samples at t = 500 s after detergent addition (Figure 2D, arrows). As expected, this treatment had no impact on the fluorescence properties of the sample in the case of dissociated mature HIV^eCFP/eYFP^ (black line). In contrast, PR digestion of membrane stripped immature particles (gray line) resulted in decay of the signal to the value observed for detergent treated mature particles, indicating dissociation of MA^eCFP^ and MA^eYFP^ following proteolytic release from the immature polyprotein.

**Figure 3:**
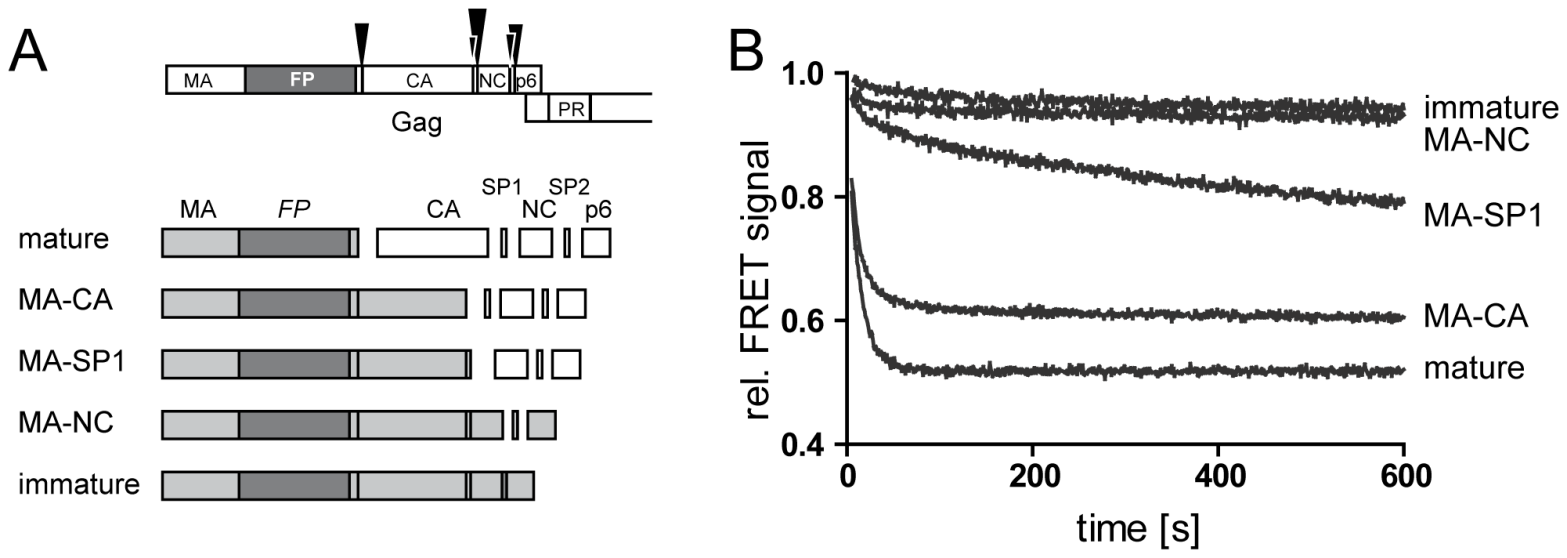
Dissociation kinetics of partially processed Gag derivatives. HIV^eCFP/eYFP^ particles containing partially processed Gag derivatives were generated by co-transfection of pCHIV, pCHIV^eCFP^ and pCHIV^eYFP^ derivatives carrying the respective mutations at individual PR processing sites in Gag. (**A**) Top, domains of the FP labeled HIV-1 Gag protein. Arrowheads indicate cleavage sites for the viral PR with the size of the arrowheads representing relative rates of cleavage (not drawn to scale). Bottom, scheme of the panel of cleavage site mutants used here. Light gray boxes represent the uncleaved Gag derived proteins comprising the FP domain (dark gray). (**B**) FRET reporter particles representing the PR cleavage site variants displayed in (A) were purified from the tissue culture supernatant of 293T cells transfected with the respective plasmid mixtures. Particles were incubated in PBS at 25°C and fluorescence emission at 528 nm was monitored (excitation: 433 nm). TX-100 (0.05%) was added at t = 0 s and incubation was continued. Intensity values shown are normalized to fluorescence intensity measured before detergent addition. Data shown are representative for measurements performed with at least four independent virus preparations.

Having established pronounced differences in the dissociation behavior of mature and immature particles as detected by this method, we investigated the dissociation properties of particles from Gag derivatives comprising different domains of the polyprotein. To this end, we generated a panel of FRET reporter viruses carrying previously characterized disabling mutations in individual PR cleavage sites within Gag, resulting in a step-wise extension of MA.FP by C-terminal Gag domains (Figure 3A). HIV^eCFP/eYFP^ particles were prepared for all variants, characterized for Gag processing by immunoblot analysis (Figure S4) and subjected to FRET measurements as described above. As shown in Figure 3B, the selected processing variants displayed distinct dissociation kinetics. Cleavage of the C-terminal SP2-p6 domain from the MA-NC layer did not significantly alter dissociation properties as compared to full-length Gag (Figure 3B, compare “immature” to “MA-NC”). Additional removal of the NC domain (“MA-SP1”) resulted in detectable destabilization of the MA-SP1 layer compared to the immature shell; nevertheless, dissociation properties were clearly distinct from those of mature particles. In contrast, the decay rate observed for MA-CA particles lacking the SP1 region resembled that of mature particles, approaching a slightly higher plateau value indicative of remaining multi- or oligomeric complexes.

**Figure 4:**
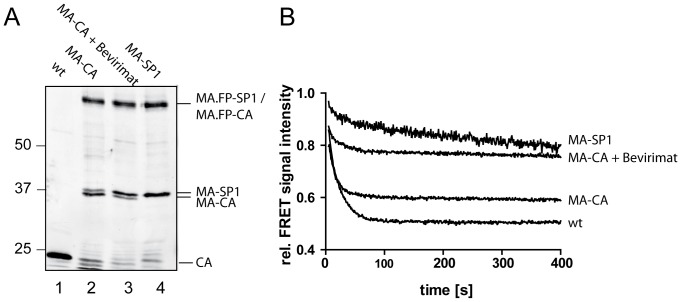
Dependence of dissociation kinetics on the relative degree of SP1 cleavage from MA-CA. (**A**) Immunoblot analysis of particles. FRET reporter particles were purified from the tissue culture supernatant of 293T cells transfected with plasmid mixtures consisting of FP-labeled and unlabeled variants of pCHIV (wt), pCHIV(MA-CA), pCHIV(MA-CA) transfected in the presence of bevirimat and pCHIV(MA-SP1), respectively. Samples of purified particles were separated by SDS-PAGE and Geg derived proteins were detected by quantitative immunoblot (LiCor Odyssey) using polyclonal rabbit antiserum raised against HIV-1 CA. Positions of molecular mass standards are indicated to the left (in kDa). (**B**) FRET measurements. The indicated particles were incubated in PBS at 25°C and fluorescence emission at 528 nm was monitored (excitation: 433 nm). At t = 0 s, 0.05% TX-100 was added and incubation was continued. Intensity values shown are normalized to fluorescence intensity measured before detergent addition.

The most striking difference was thus observed between the MA-CA and MA-SP1 variants that differ only in a 14 amino acid peptide at the C-terminus of CA. Close similarity between the observed dissociation properties of MA and MA-CA layers indicates a minor contribution of the CA domain to oligomer stability upon SP1 removal. In contrast, the much slower dissociation kinetics observed for MA-SP1 indicates a pronounced oligomer stabilizing effect exerted by the presence of SP1. Uncleaved MA-SP1 molecules detected in the MA-CA virus preparations used (see Figure 4, lane 2) may thus explain the residual FRET signal detected upon completion of MA-CA particle dissociation. While MA-SP1 does not represent a *bona fide* intermediate of HIV-1 maturation due to more rapid cleavage at MA-CA compared to CA-SP1, we assume that the stabilizing effect of SP1 on the CA lattice can also manifest itself in the context of CA-SP1 assemblies.

To further characterize the influence of SP1 on the dissociation properties we performed comparative experiments based on drug-induced inhibition of processing. The HIV-1 inhibitor bevirimat specifically impairs PR mediated processing at the CA-SP1 cleavage site by binding to the Gag substrate [31]–[34]. Use of this compound allowed us to prepare particles containing elevated levels of MA-SP1 in the absence of a mutation at the CA-SP1 cleavage site. Since bevirimat treatment of virus producing cells does not completely block CA-SP1 processing [33], the degree of cleavage in the resulting particle preparations was determined by immunoblot (Figure 4A).Preparations of the MA-CA variant contained mainly MA-CA, but also traces of uncleaved MA-SP1 (Figure 4A, lane 2). A comparable impairment of CA-SP1 processing by blocking cleavage at the upstream MA-CA processing site has been observed previously in the case of unlabeled HIV-1 [35]. As expected, only MA-SP1 was detected in the case of MA-SP1 mutant particles (Figure 4A, lane 4). In contrast, MA-CA particles produced in the presence of bevirimat displayed an intermediate phenotype with respect to CA-SP1 processing (Figure 4A, lane 3) that was also reflected in the results from FRET measurements (Figure 4B). Whereas a more rapid initial dissociation was observed for MA-CA particles produced in the presence of bevirimat compared to particles containing exclusively MA-SP1, an elevated plateau value of ∼75% of the initial value in both cases indicated the presence of significant amounts of residual detergent stable complexes. The latter finding may indicate a stabilizing effect of bevirimat beyond the degree corresponding to the proportion of uncleaved CA-SP1 molecules. This interpretation is consistent with recent structural data from Keller et al. [36], who reported that the CA-SP1 layer is retained in an immature-like lattice conformation in the presence of bevirimat.

These measurements provided semi-quantitative information on dissociation rates. Kinetic analysis of FRET data was hampered, however, by a loss of information on the initial rapid phases of the dissociation process due to the manual mixing procedure. Therefore we performed stopped-flow measurements that allow fast, automated mixing of virion suspension and detergent solution with signal detection on the millisecond time scale. The setup used provided the additional advantage that static light scatter and FRET signals could be measured simultaneously. To confirm that elevated pressure under rapid flow conditions did not result in physical disruption of particles, control experiments were carried out using HIV^eCFP/eYFP^ in PBS without TX-100 (Figure S5). A stable signal over time was observed in static light scatter as well as in FRET measurements, confirming that particle disruption did not occur in the absence of detergent.

**Figure 5.Comparison:**
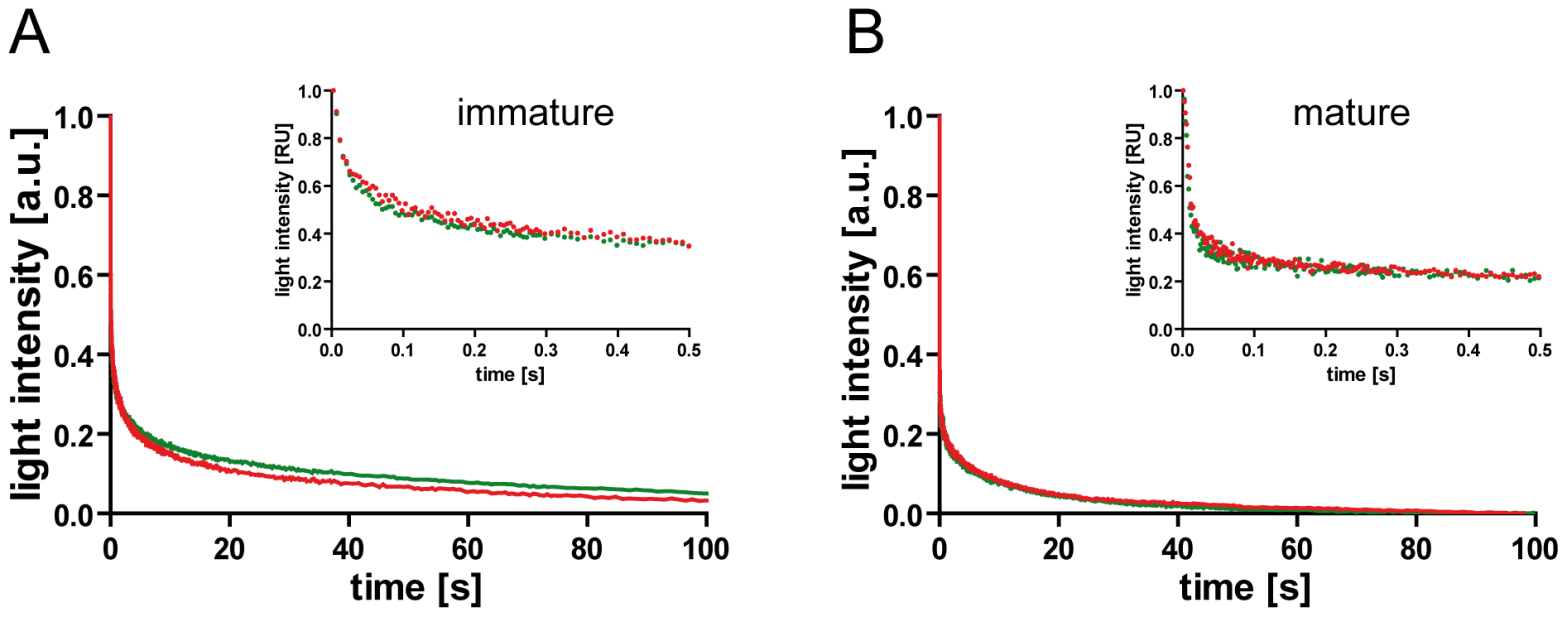
of HIV^eCFP/eYFP^ with unlabeled HIV-1 particles by light scatter analysis. Static light scatter intensities of HIV (red) or HIV^eCFP/eYFP^ (green) particle preparations were analyzed by stopped-flow measurement in PBS/0.05% TX-100. Graphs show data obtained for immature (**A**) and mature (**B**) particles, respectively. For a direct comparison of curves, data were normalized for the amplitude of the respective data set (1 =  initial value, 0 =  plateau reached at 300 s). Curves shown represent averaged data from 5–6 individual measurements. Main graphs show the first 100 s of the dissociation reaction, smaller insets display enlargements of the initial phases (500 ms). Data shown are representative for measurements performed with at least two independent virus preparations.

Using this setup, we again compared immature and mature HIV^eCFP/eYFP^ particles with variants MA-SP1 and MA-CA. While the complex context used here clearly limits the mechanistic interpretation of data from light scatter experiments, these measurements allowed us to validate reporter particles carrying an additional FP moiety inserted in ∼50% of the Gag molecules against unlabeled particles derived from HIV-1 Gag alone. As shown in Figure 5A, identical decay curves were obtained upon detergent addition when comparing immature HIV^eCFP/eYFP^ particles to their unlabeled counterpart. The same was true for a comparison of mature labeled and unlabeled particles, respectively, (Figure 5B), confirming that the presence of FP domains did not affect dissociation properties. Furthermore, the finding that decay curves were nearly identical for labeled and unlabeled particles of the same maturation status, while differing between immature and mature preparations suggested that decay curve characteristics measured here mainly reflected the maturation status of the virus particles rather than unspecific impurities or aggregates contained in individual preparations. High temporal resolution light scatter decay curves obtained for the different HIV^eCFP/eYFP^ processing variants (Figure S6) were dominated by at least one very rapid phase, which escaped exact measurement even at the ms time resolution of stopped-flow experiments. However, even on this scale immature particles were characterized by substantially lower initial dissociation rates compared to mature particles (Figure S6). Particles dissociated with highly complex kinetics, involving one or more rapid phase(s) and a slower phase, with 90% completion reached within less than 5 min. Assignment of individual kinetic phases was hampered by the fact that traces could not be fitted even to higher order exponential equations. Good fits to the experimental data were only obtained using stretched exponentials, suggesting a multitude of processes. This high degree of complexity may be explained by a mixture of individual particles following alternate dissociation pathways.

**Figure 6:**
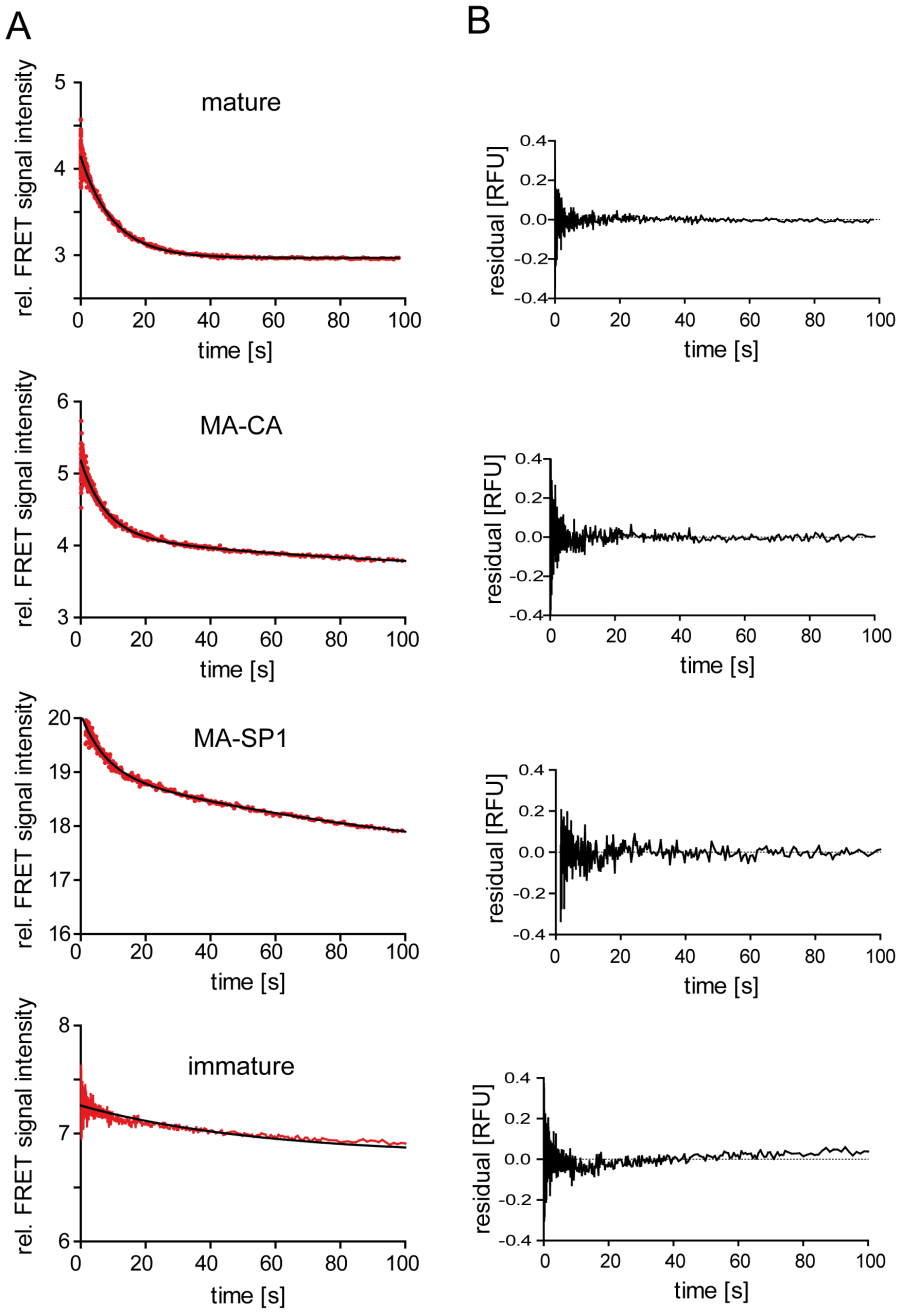
Stopped-flow FRET measurements. (**A**) The indicated HIV^eCFP/eYFP^ reporter particles were purified from the supernatant of transfected 293T cells and suspended in PBS. Dissociation in the presence of 0.05% TX-100 was monitored by FRET analysis using a stopped-flow setup as described in materials and methods. FRET signal intensities averaged from six individual measurements each are plotted in red; black lines indicate single (mature, immature) or double (MA-CA, MA-SP1) exponential fits to the data, yielding the kinetic constants summarized in Table 1. (**B**) Residual plots derived from the data shown in (A) are represented besides the respective graphs.

FRET signal intensities were monitored in parallel to the light scatter recordings. Whereas light scatter measurements are sensitive to changes in the overall composition of the sample from poly- or oligomeric units of various sizes, FRET only detects disruption of contacts between neighboring molecules in the lattice. Decay curves of the FRET signal determined for mature, MA-CA, MA-SP1 and immature particles, respectively, are shown in Figure 6A. Comparison of FRET and light scatter data of the respective mutants showed that one or more very rapid phase(s) detected by light scatter in all cases was not reflected in the corresponding FRET measurements. Contribution of rapid processes to light scatter decay was also observed for preparations of immature particles (Figure S6, ‘immature’), that displayed only a minor decrease in FRET intensity in the cuvette-based measurements (compare Figures 2 and 3) and still showed complete shells and large Gag assemblies following detergent stripping as assessed by qualitative EM (Figure 1). It is thus reasonable to assume that the rapid phases observed in light scatter measurements represent mainly breakage of the semi-spherical Gag shell into large fragments. Cyro-EM analyses of immature HIV-1 particles indicate that the Gag shell is incomplete and curvature of the hexameric Gag lattice is accomplished by irregular defects [8], [37], [38] that may present pre-determined breaking points in the overall shell architecture.

As shown in Figure 6A and Table 1, FRET decay curves recorded for mature and immature particles, respectively, followed single exponential kinetics, whereas decay curves of MA-CA and MA-SP1 particles were more complex and best fits were obtained using a double exponential equation. Fast and slow rate constants were similar for both variants, but the relative amplitudes differed, with a higher contribution of the rapid phase in the case of MA-CA (Table 1). The mature MA shell displayed only one fast FRET decay phase, pointing to comparable strengths of contacts between individual MA-FP monomers. The determined rate was similar to that of the slower phase determined by light scatter measurements for mature particles, indicating that this phase may reflect dissociation to small MA oligomers or monomers. Spectral analysis (compare Figure 2C) suggested that the final decay product from this phase represents mainly monomeric MA.FP subunits in the case of mature particles. Although calculation of rates was precluded by the complex kinetics, our measurements indicate that MA.FP layer dissociation is a gradual process reaching 90% completion within less than 5 min under the conditions used (Figures 2 and 3). In contrast, live-cell imaging of individual fusion events using similar fluorescent virus derivatives indicated that strong MA.FP signals remained associated with viral cores for several minutes following loss of the viral envelope [39]. Whether this difference is due to subtle impairment of Gag maturation through incorporation of FP-tagged Vpr protein in the viruses used for imaging [26] or reflects differences between the crowded cytoplasmic environment and conditions in solution remains to be determined.

**Table 1:** Rate constants derived from best exponential fits to the dissociation data shown in Figure 6.

	mature	MA-CA	MA-SP1	immature
type	single exponential	double exponential	double exponential	single exponential
Y (initial)	4.14	5.18	20.07	7.27
k1 [s-1]	0.099	0.127	0.129	0.018
amplitude 1	1.17	1.04 (62.7%)	1.07 (34.5%)	0.46
k2 [s-1]	n.a.	0.0082	0.0078	n.a.
amplitude 2	n.a.	0.62 (37.3%)	2.03 (65.5%)	n.a.
offset	2.97	3.52	16.97	6.79

Amplitudes are indicated as absolute values [a.u.]; values in brackets indicate percentage of total amplitude. N.a., not applicable.

Dissociation properties of the immature and partially processed Gag derivatives are expected to be governed by the hexameric immature CA lattice. Based on the lattice dimensions determined by cryo-electron tomography [7], [8] and an R_0_ value of 4.92 nm for the eCFP-eYFP FRET pair [40], and with the simplifying assumption that eCFP-, eYFP- and unlabeled Gag molecules are homogeneously distributed, it can be estimated that four labeled Gag molecules are within transfer distance to any given labeled monomer. Two of these belong to the same hexameric subunit, while two others are inter-hexagonal neighboring molecules of the interface triangle. Molecules in the next distance shell (5–9 nm), which also contribute with lower transfer efficiency to the observed FRET signal, comprise ∼2 intra- and 6 inter-hexagonal neighbors. Accordingly, ∼2/3 of the FRET signal should vanish by separation between individual hexamers, while ∼1/3 of signal decay would be contributed by hexamer disruption in the case of complete dissociation. The single slow exponential FRET decay phase observed for immature particles is consistent with disruption of only one type of contacts, most likely inter-hexameric. This is consistent with cuvette-based FRET measurements showing only a very minor decay in overall FRET signal for immature particles (compare Figures 2 and 3), indicating that the dissociation processes did not reach the level of small oligomers or monomers.

Light scatter decay rates of MA-CA and MA-SP1 variants did not differ significantly (Figure S6). We therefore presume that the different dissociation behavior observed in our initial FRET measurements (Figure 3) is manifested mainly at the level of oligomer dissociation. Rapid FRET decay curves for the partially processed MA-CA and MA-SP1 variants were more complex than those of mature and immature particles and best fits to the experimental data were obtained using double exponential equations (Figure 6 and Table 1). The relative ratio of amplitudes in the range of about 1/3 to 2/3 observed is consistent with the assignment of the two phases to dissociation of inter- and intrahexameric contacts displaying different interaction strengths. Accordingly, slightly different relative ratios of these two phases for the two virus variants would imply differences in distribution in terms of inter- versus intra-hexagonal contacts.

In summary, the system developed allowed us to study dissociation rates of HIV-1 particles formed in virus producing cells and containing components of the authentic virus with high temporal resolution. While the complex dissociation kinetics displayed by these molecular assemblies, as well as the presence of the additional FP domain required for readout, prevented a definitive assignment of kinetic rate constants to individual dissociation steps, our measurements provided general information on the process. In particular, they highlight a strongly lattice-stabilizing function of the SP1 peptide. Cryo-electron tomography of the immature HIV-1 lattice revealed rod-like structures connecting the CA C-terminal domain with the NC layer [8], [37]. While not sufficiently resolved in current structural models, these features were hypothesized to represent six-helix bundles of α-helices comprising C-terminal residues of CA and the SP1 region [8], whose destruction by proteolytic processing could destabilize immature lattice interactions and promote formation of the mature assembly. Comparison of tomographic reconstructions of MA-CA and MA-SP1 shells did not reveal significant structural differences in the CA layer [35]. However, data reported here clearly indicate that the SP1 region, which was not resolved in the tomogram of MA-SP1, significantly contributes to the stability of intermolecular interactions in the immature lattice. Analogous to the *in vitro* measurement of CA assembly rates [14], the system developed here allows quantitative analyses of the effects of mutations or small molecules on the HIV-1 disassembly process and will thus be useful for further studies of Gag intermolecular interactions.

## Supporting Information

Figure S1 **Decay of light scattering signal upon detergent mediated particle dissociation.** 293T cells were transfected with pCHIV and grown in the absence (mature, green line) or presence (immature, red line) of 2 µM HIV-1 protease inhibitor LPV. In parallel, cells were transfected with the empty vector pCDNA3.1 (Zeo) as a mock control. At 44 h.p.t., supernatants were harvested and particles were enriched by ultracentifugation through a 20% (w/w) sucrose cushion. Samples corresponding to 3.2 ml of tissue culture supernatant each were resuspended in PBS and equilibrated at 25°C. Light scatter intensities were determined using an SLM AB2 spectrofluorometer at a wavelength of 436 nm, using identical instrument settings for all measurements. At t = 0, TX-100 was added to a final concentration of 0.05% and measurement was continued at 25°C for 10 min. **Red**, immature; **green**, mature; **black**, mock particle preparation; **blue**, PBS buffer control. Data from mature and immature particles, respectively, normalized to the scatter intensity before detergent addition, are displayed in Figure 1B.
(TIF)

Figure S2 **Dual labelling of HIV-1^CFP/YFP^ reporter particles.** Dual labeled particles were purified from the supernatant of transfected 293T cells as descriden in materials and methods. Particles were suspended in PBS, adhered to the glass bottom of LabTek chamber slides and imaged by spinning disc confocal microscopy in the CFP and YFP channel.
(TIF)

Figure S3 **Time dependence of FRET signal intensity changes upon addition of different detergent concentrations.** HIV^eCFP/eYFP^ particles were incubated in PBS at 20°C and excited at 433 nm. Fluorescence emission was recorded at a wavelength of 528 nm. At t = 0 s, the viral envelope was disrupted by addition of TX-100 to a final concentration of 0.05%, 0.1% or 0.2%, respectively. Data were normalized to the fluorescence intensity measured before detergent addition.
(TIF)

Figure S4 **Immunoblot analysis of partially processed particles used in the experiment shown in main** **Figure 3** **.** Particles were generated by co-transfection of pCHIV, pCHIV^CFP^ and pCHIV^YFP^ (2∶1∶1), respectively, carrying mutations at a subset of PR processing sites in Gag as illustrated in Figure 3A. Particles were purified from the tissue culture supernatant of 293T cells transfected with the respective pCHIV derived plasmid mixtures by ultracentrifugation through a 20% (w/w) sucrose cushion. Samples were separated by SDS-PAGE and proteins were transferred to a nitrocellulose membrane. Gag derived products were detected by quantitative immunoblot (LiCor) using polyclonal rabbit antisera reaised against the indicated HIV-1 proteins. Positions of molecular mass standards (in kDa) are indicated to the left.
(TIF)

Figure S5 **Effect of high flow rates on particle dissociation.** Static light scatter (**A**) and FRET (**B**) signal intensities of mature HIV^eCFP/eYFP^ particles were analyzed by stopped-flow measurements in PBS without the addition of TX-100. Measurements were performed at 25°C. Initial values were set to 1.
(TIF)

Figure S6 **Stopped-flow light scatter measurements.** (**A**) The indicated HIV^eCFP/eYFP^ reporter particles were purified from the supernatant of transfected 293T cells and suspended in PBS. Dissociation in the presence of 0.05% TX-100 was monitored by light scatter analysis using a stopped-flow setup as described in materials and methods. Data shown represent averages from 6 individual measurements for each variant. (**B**) Expansion of the graphs shown in A, displaying the initial 500 ms.
(TIF)
