# Supplementary figures and images for: In Vitro Analysis of Human Immunodeficiency Virus Particle Dissociation: Gag Proteolytic Processing Influences Dissociation Kinetics
Source: PLoS One. 2014 Jun 10;9(6):e99504. doi: 10.1371/journal.pone.0099504 (PMC4051761; doi:10.1371/journal.pone.0099504)

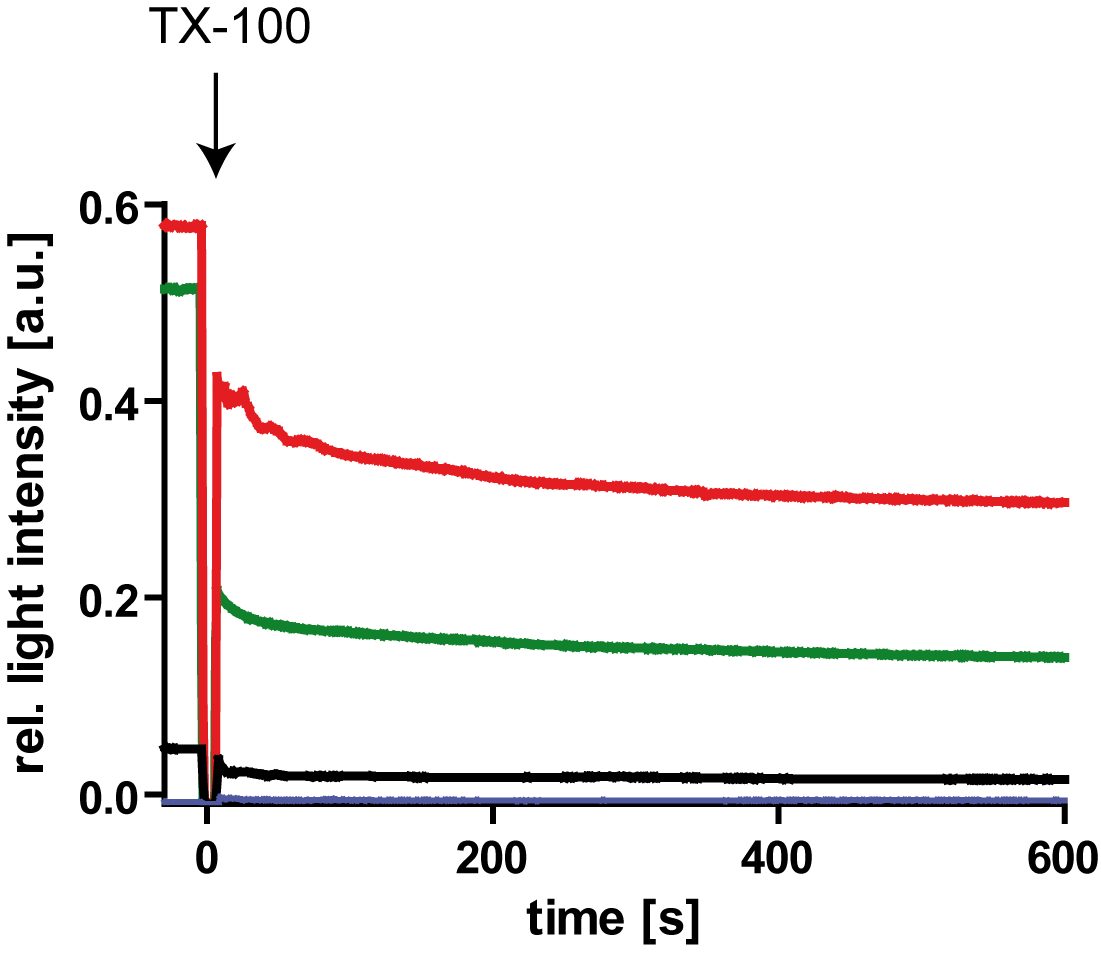

Supplement: Figure S1 — Decay of light scattering signal upon detergent mediated particle dissociation. 293T cells were transfected with pCHIV and grown in the absence (mature, green line) or presence (immature, red line) of 2 µM HIV-1 protease inhibitor LPV. In parallel, cells were transfected with the empty vector pCDNA3.1 (Zeo) as a mock control. At 44 h.p.t., supernatants were harvested and particles were enriched by ultracentifugation through a 20% (w/w) sucrose cushion. Samples corresponding to 3.2 ml of tissue culture supernatant each were resuspended in PBS and equilibrated at 25°C. Light scatter intensities were determined using an SLM AB2 spectrofluorometer at a wavelength of 436 nm, using identical instrument settings for all measurements. At t = 0, TX-100 was added to a final concentration of 0.05% and measurement was continued at 25°C for 10 min. Red, immature; green, mature; black, mock particle preparation; blue, PBS buffer control. Data from mature and immature particles, respectively, normalized to the scatter intensity before detergent addition, are displayed in Figure 1B. (TIF) [file pone.0099504.s001.tif]

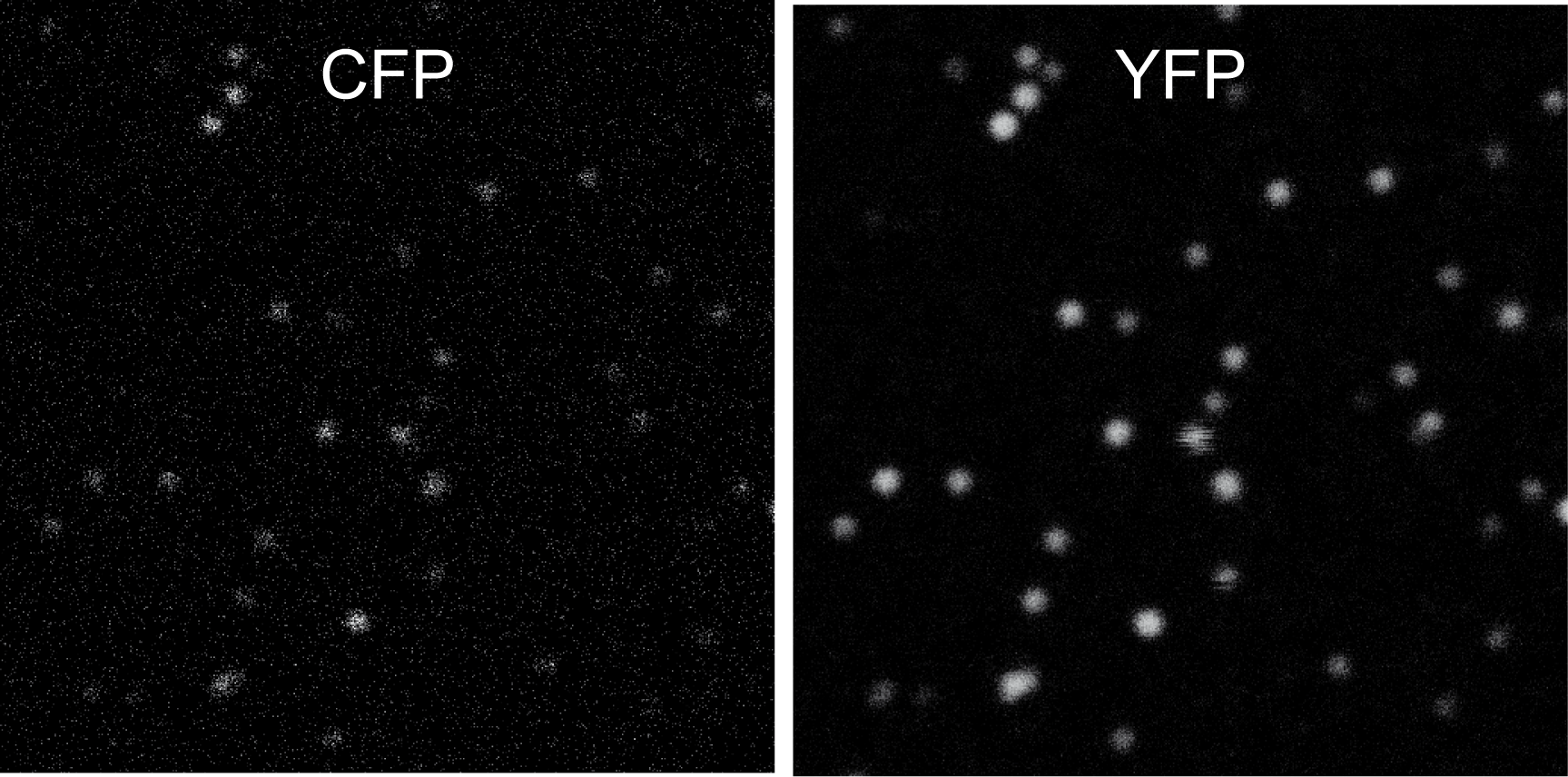

Supplement: Figure S2 — Dual labelling of HIV-1CFP/YFP reporter particles. Dual labeled particles were purified from the supernatant of transfected 293T cells as descriden in materials and methods. Particles were suspended in PBS, adhered to the glass bottom of LabTek chamber slides and imaged by spinning disc confocal microscopy in the CFP and YFP channel. (TIF) [file pone.0099504.s002.tif]

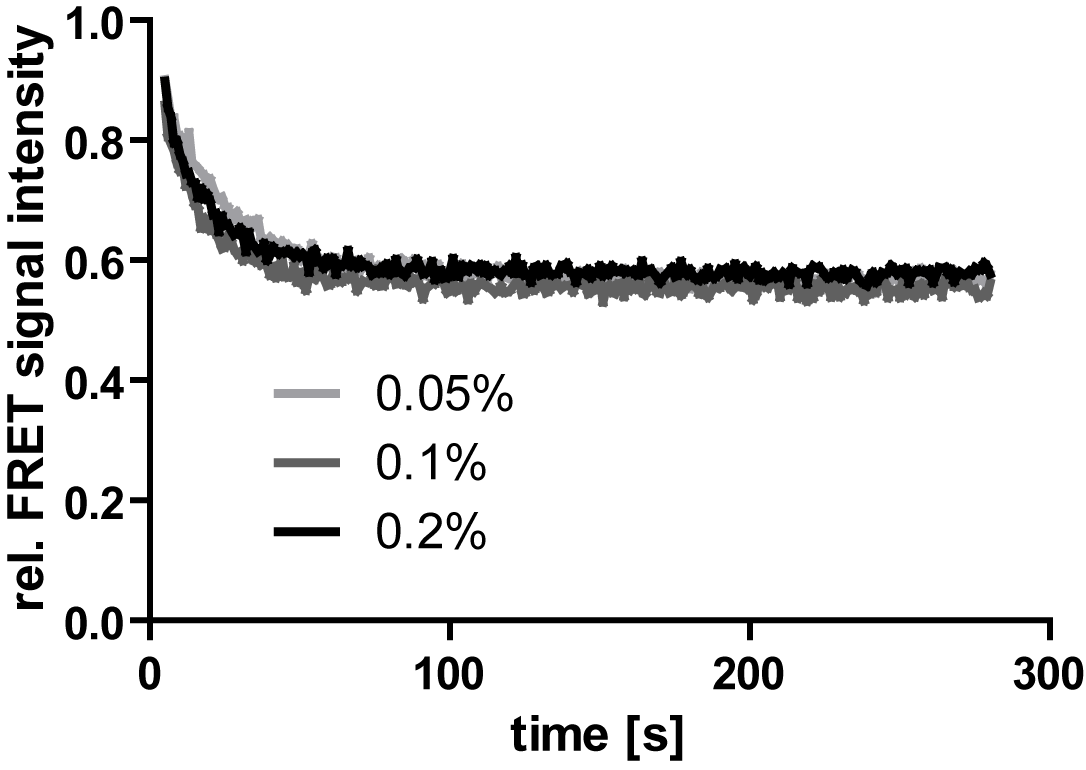

Supplement: Figure S3 — Time dependence of FRET signal intensity changes upon addition of different detergent concentrations. HIVeCFP/eYFP particles were incubated in PBS at 20°C and excited at 433 nm. Fluorescence emission was recorded at a wavelength of 528 nm. At t = 0 s, the viral envelope was disrupted by addition of TX-100 to a final concentration of 0.05%, 0.1% or 0.2%, respectively. Data were normalized to the fluorescence intensity measured before detergent addition. (TIF) [file pone.0099504.s003.tif]

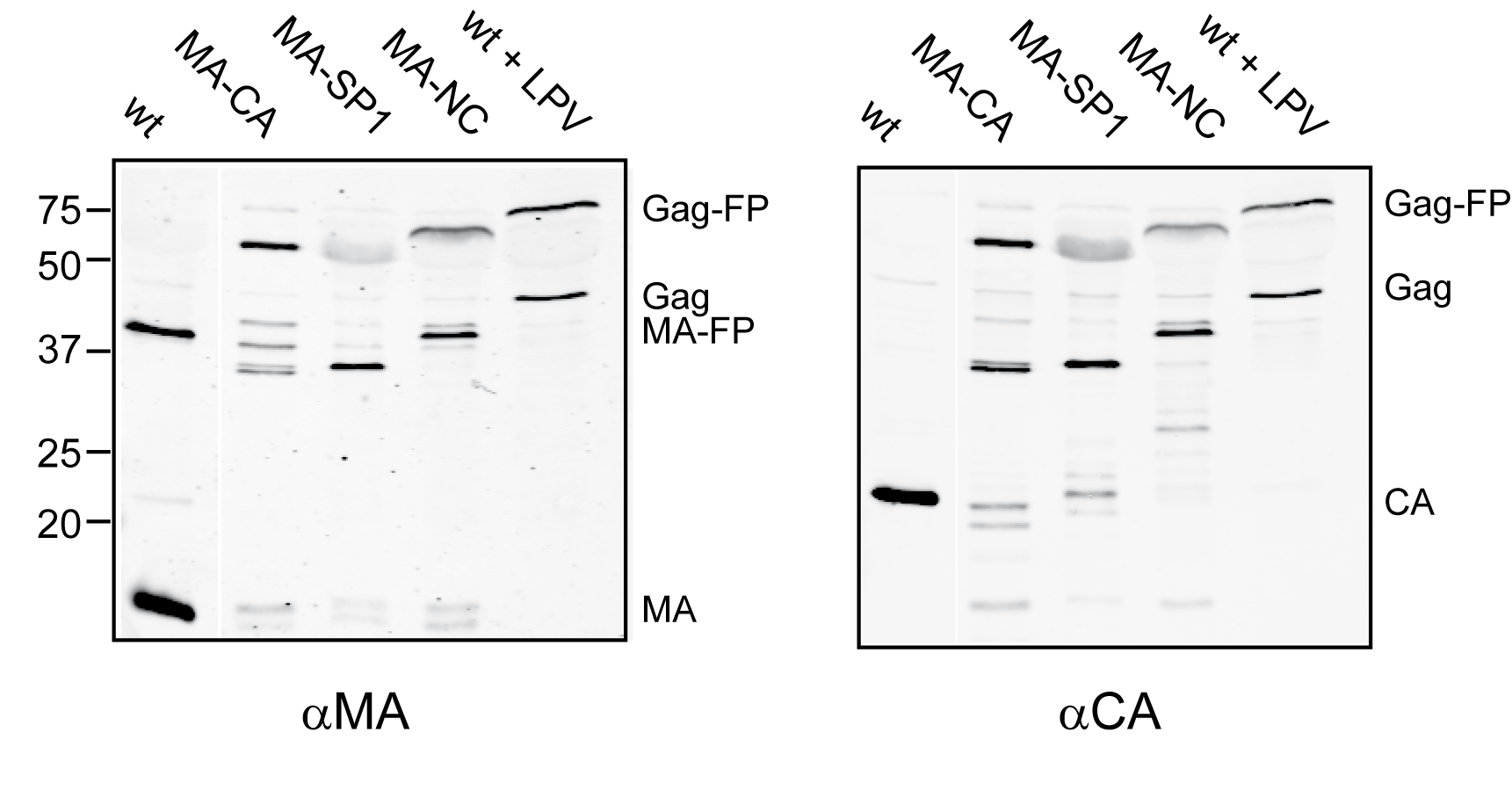

Supplement: Figure S4 — Immunoblot analysis of partially processed particles used in the experiment shown in main Figure 3 . Particles were generated by co-transfection of pCHIV, pCHIVCFP and pCHIVYFP (2∶1∶1), respectively, carrying mutations at a subset of PR processing sites in Gag as illustrated in Figure 3A. Particles were purified from the tissue culture supernatant of 293T cells transfected with the respective pCHIV derived plasmid mixtures by ultracentrifugation through a 20% (w/w) sucrose cushion. Samples were separated by SDS-PAGE and proteins were transferred to a nitrocellulose membrane. Gag derived products were detected by quantitative immunoblot (LiCor) using polyclonal rabbit antisera reaised against the indicated HIV-1 proteins. Positions of molecular mass standards (in kDa) are indicated to the left. (TIF) [file pone.0099504.s004.tif]

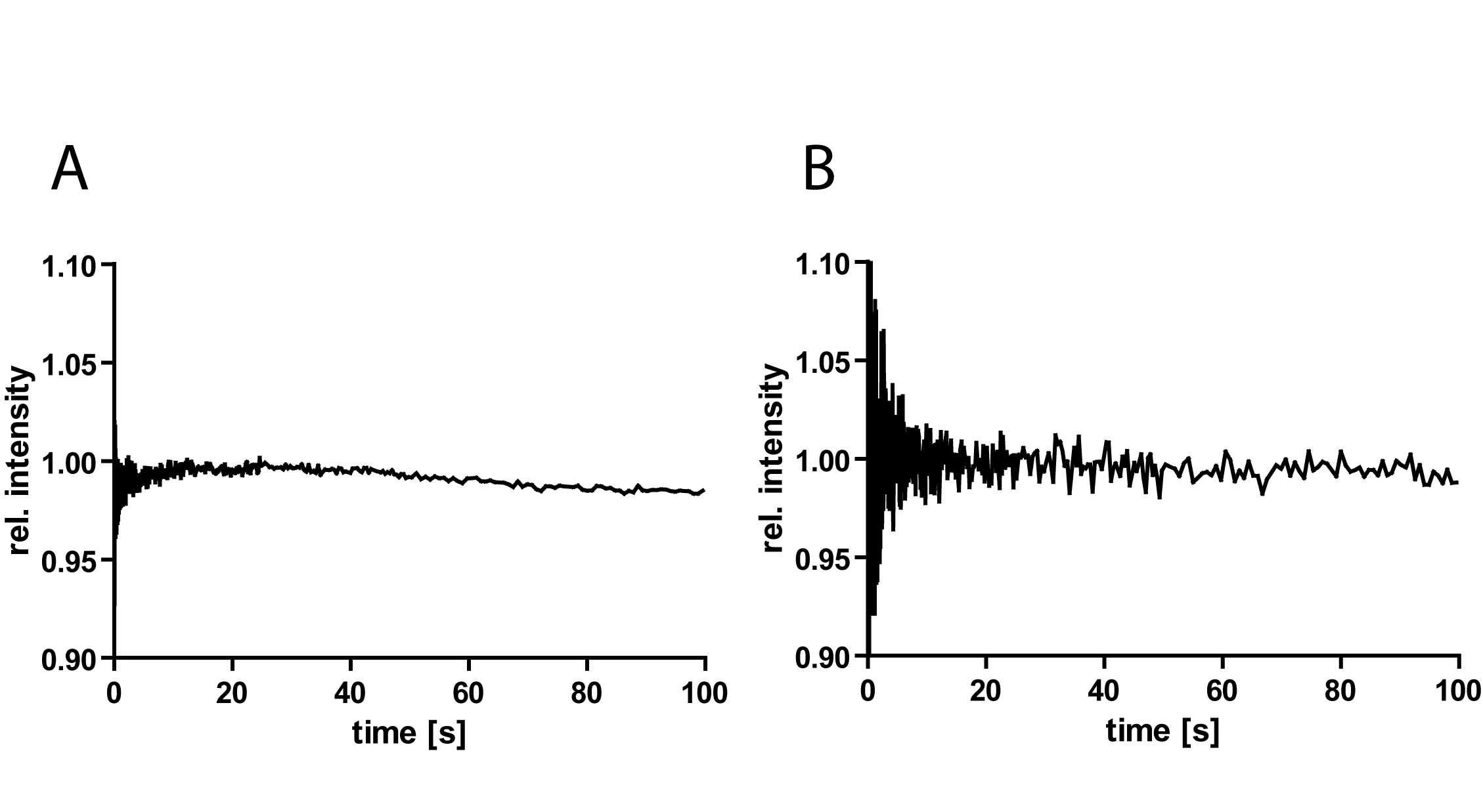

Supplement: Figure S5 — Effect of high flow rates on particle dissociation. Static light scatter (A) and FRET (B) signal intensities of mature HIVeCFP/eYFP particles were analyzed by stopped-flow measurements in PBS without the addition of TX-100. Measurements were performed at 25°C. Initial values were set to 1. (TIF) [file pone.0099504.s005.tif]

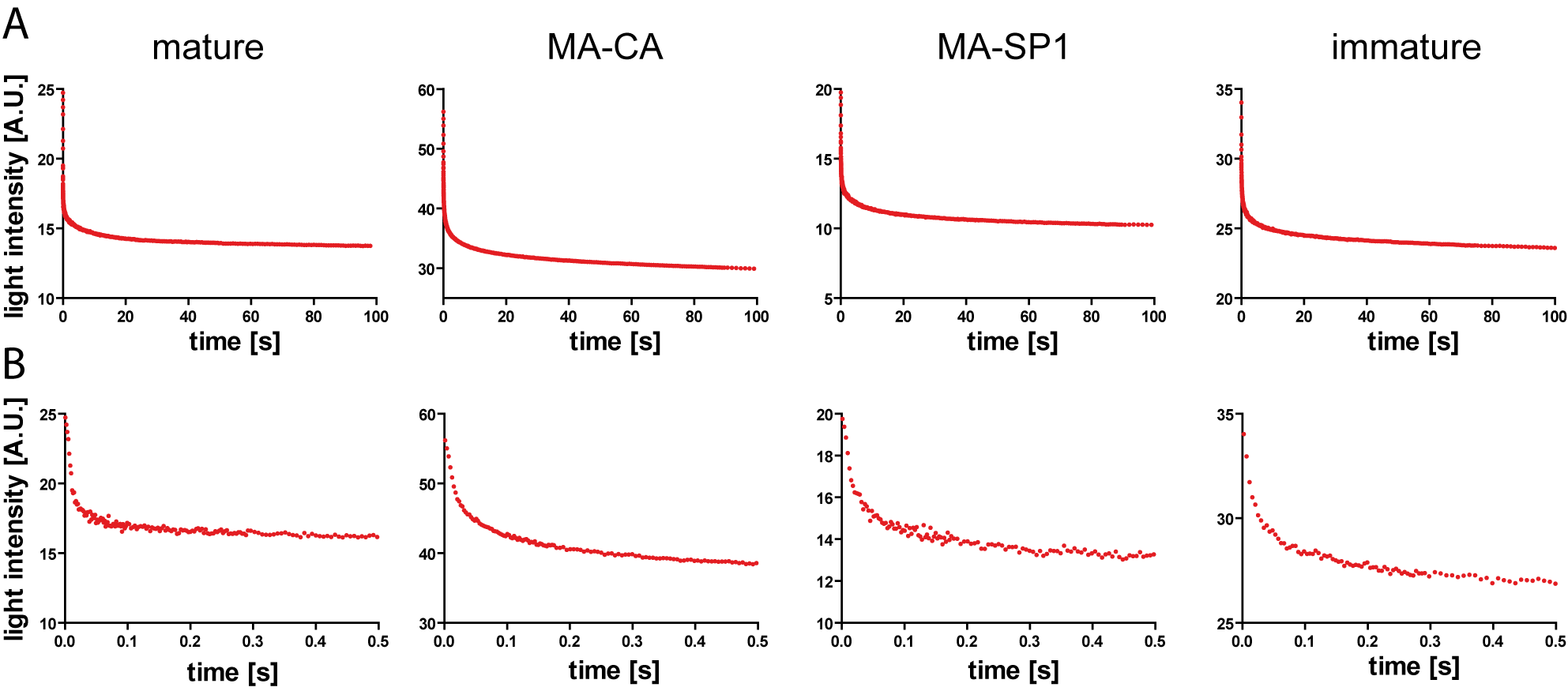

Supplement: Figure S6 — Stopped-flow light scatter measurements. (A) The indicated HIVeCFP/eYFP reporter particles were purified from the supernatant of transfected 293T cells and suspended in PBS. Dissociation in the presence of 0.05% TX-100 was monitored by light scatter analysis using a stopped-flow setup as described in materials and methods. Data shown represent averages from 6 individual measurements for each variant. (B) Expansion of the graphs shown in A, displaying the initial 500 ms. (TIF) [file pone.0099504.s006.tif]
